# Erratum

**DOI:** 10.1155/1990/91303

**Published:** 1990

**Authors:** J. D. Greig

**Affiliations:** University Department of Surgery Royal Infirmary of Edinburgh Lauriston Place Edinburgh EH3 9YW Scotland


					226 HPB NEWS
THE FIRST ASIAN CONFERENCE OF HPB SURGERY
The First Asian Conference of HPB Surgery will take place at Central Plaza Hotel
in Bangkok, Thailand on January 10 to 12, 1991.
Specific topics to be covered are
1. Biliary Calculi including Hepatolithiasis
2. Biliary Infection
3. Hepatocellular Carcinoma
4. Acute Pancreatitis
5. Ideal techniques in HPB Surgery
6. Others Video and Plenary sessions
Deadline for abstract: September 30, 1990
Official language, English
Organizing Committee
R. Tsuchiya, Y. Saito, F. Hanyu, Advisors; T. Takada, Director; T. Yamakawa,
Co-director; T. Nakayama, K. Satake, Y. Kawarada, T. Nagakawa, K. Takasaki,
M. Ryu, Y. Nimura, Y. Ogura, Scientific advisors.
Additional information
contact T. Takada, M.D., F.A.C.S.
1st Department of Surgery,
Teikyo University School of Medicine,
2-11-1, Kaga, Itabashi-ku, Tokyo 173 Japan
Fax: 03-962-2128
ERRATUM
Letter to the Editor Vol.1, pp. 257-258
This letter, from J.D. Greig and J.R. Goldring, appeared with incomplete infor-
mation for those wishing to contact the authors.
The letter concerned the paper by Andersson and colleagues entitled "Haemo-
peritoneum after spontaneous rupture of liver tumor: results of surgical treat-
ment". HPB Surgery 1988:1:81-83.
Please note that any correspondence, or any requests for the letter should be
addressed to:
Mr. J.D. Greig
University Department of Surgery
Royal Infirmary of Edinburgh
Lauriston Place
Edinburgh EH3 9YW

				

